# Collagen Type III and VI Turnover in Response to Long-Term Immobilization

**DOI:** 10.1371/journal.pone.0144525

**Published:** 2015-12-07

**Authors:** Shu Sun, Kim Henriksen, Morten A. Karsdal, Inger Byrjalsen, Jörn Rittweger, Gabriele Armbrecht, Daniel L. Belavy, Dieter Felsenberg, Anders F. Nedergaard

**Affiliations:** 1 Nordic Bioscience Biomarkers and Research, Herlev, Denmark; 2 Institute of Sports Medicine Copenhagen, Bispebjerg Hospital, Copenhagen NW, Denmark; 3 Institute of Aerospace Medicine, German Aerospace Center, Köln, Germany; 4 Center for Muscle and Bone Research, Charité Universitätsmedizin Berlin and Free and Humboldt Universities, Berlin, Germany; 5 Deakin University, Centre for Physical Activity and Nutrition Research, School of Exercise and Nutrition Sciences, Burwood, Victoria, 3125, Australia; University of Bergen, NORWAY

## Abstract

**Background:**

Muscle mass and function are perturbed by immobilization and remobilization. When muscle mass changes, the quality and quantity of the extracellular matrix protein, particularly the collagens, change with it. In this study, we investigated the temporal profile of three peptide biomarkers derived from turnover of collagen type III and type VI in a long-term immobilization and remobilization study. We also compared individual biomarker levels with Lean body Mass (LBM) and changes therein, hypothesizing that these biomarkers would be biomarkers of the remodeling processes associated with immobilization and/or remobilization.

**Methods:**

In the Berlin bed rest study, 20 young men were recruited and randomly assigned to 8-week’s strict bed rest with or without resistive vibration exercise countermeasure. We measured three neo-epitope ELISA kits in the serum samples of this study: Pro-C3, measured the synthesis of collagen type III; Pro-C6, measured the synthesis of collagen type VI; and C6M measured the degradation of collagen type VI induced by MMP-2 and MMP-9 cleavage.

**Results:**

Pro-C3 and Pro-C6 biomarkers are up-regulated with both immobilization and remobilization, whereas C6M is hardly affected at all. We found that Pro-C3 and C6M levels are related to LBM at baseline and that high levels of Pro-C6 are associated with smaller changes in muscle mass during both immobilization and remobilization.

**Conclusion:**

The Pro-C3 and–C6 biomarkers change likely reflect remodeling changes in response to unloading or reloading, whereas C6M does not appear to respond to unloading. Pro-C3 and C6M levels correlate with LBM at baseline, while Pro-C6 is related to the anabolic and catabolic responses to unloading and reloading.

## Introduction/Background

Muscle mass and function are reduced with age, along with a range of pathologies and inactivity. It is reported that elderly men lose 1% leg lean mass per year [1], that 2–3% of muscle mass is lost per week during immobilization [2] and sometimes even quicker with cachexia [3]. Impaired muscle mass and function in elderly or hospitalized individuals has been shown to be associated with (co)morbidity and mortality [4]. With the population age increasing in the industrialized world, maintaining functional independence shows increasing importance. Nowadays, the diagnosis and monitoring of muscle loss rely on the imaging examination, e.g. magnetic resonance imaging (MRI), computed tomography (CT) and dual-energy X-ray absorptiometry (DXA) [4]. However, such examinations are not in widespread clinical use, probably owing to its expensive cost and inconvenience to be used in routine clinical care. Urinary and serological biomarkers such as creatinine and 3-methyl histidine have also been shown to be able to assess muscle mass and could thus assist the management of muscle loss. However, high variation and poor validity of these assays limit their use [5]. In summary, there is an urgent need for biomarkers which can be used in the diagnosing and predicting muscle function as well as monitoring anticatabolic and anabolic treatment outcomes [6].

Loss of muscle mass is driven by unbalanced turnover of muscle extracellular proteins [7, 8]. As protein turnover, particularly of extracellular proteins, can allow proteolytic fragments to escape into the circulation, quantitative or qualitative changes in protein metabolism can give rise to biomarker profiles that can be of use in monitoring muscle mass or function [5].

Collagens are important extracellular proteins of skeletal muscle, which could contribute to the passive tension of muscle [9]. Collagen type III is expressed in most of the collagen type I containing tissues except for bone, and is an important component of connective tissues, muscle tissues and skin [10]. PIIINP is the N-terminal propeptide of collagen type III, which is removed during mature collagen type III synthesis [11]. It has been reported to be related to the anabolic response of hormone treatment [12, 13]. Recently, a new ELISA kit was developed by applying monoclonal antibody targeting the N-protease cleavage site of collagen type III propeptide, which could assess the true synthesis of collagen type III [14].

Collagen type VI is a unique extracellular collagen, which can form an independent microfibrillar network in the basement membrane of cells. It can interact with other matrix proteins including collagens, biglycan, proteoglycans, *etc* [15–17]. In muscle, collagen type VI is part of the sarcolemma and involved in anchoring the muscle fiber into the intramuscular extracellular matrix, thus involved in force transmission [18, 19]. Moreover, mutations in collagen type VI can cause Bethlem myopathy and Ullrich congenital muscular dystrophy [20]. It has been reported that the C-terminus of collagen type VI α3 chain is cleaved off from the mature type VI microfibril after secretion [21, 22]. Therefore, the level of C-terminus of α3 chain could reflect the level of newly formed mature collagen type VI. In order to investigate the synthesis of collagen type VI, we developed a Pro-C6 ELISA kit targeting the C-terminus of α3 chain. In addition, collagen type VI is also a substrate of MMPs [23]. Previous studies showed that both MMP-2 and MMP-9 are relevant to muscle atrophy [24, 25]. Therefore, collagen type VI degradation fragment by MMP-2 and MMP-9 could be interesting in relation to immobilization induced muscle atrophy.

In this study, we measured three neo-epitope biomarkers Pro-C6 (measuring the C-terminus α3(VI) chain) and C6M (measuring collagen type VI fragment degraded by MMP-2 and MMP-9) [23], and Pro-C3 (measuring the synthesis of collagen type III) [14], which directly measure the turnover of collagen type III and VI in the Berlin bed rest study–using bed rest immobilization and remobilization as a human model of muscle atrophy and hypertrophy.

## Materials and Methods

### Bed rest study

The Berlin bed rest study (BBR) has been described elsewhere [26, 27]. Briefly, 20 healthy young men were recruited and underwent a strict 8-week bed rest study. The 20 young men were then randomly divided into two groups. The resistive vibration exercise group (RVE) group was assigned to resistive vibration exercise 11 times per week. The resistive vibration exercises were performed with a vibration exercise apparatus at the end of the beds, and with the subject pulling themselves towards the vibration plate through waist and shoulder straps and handles. The control group (CTRL) was not allowed to perform any exercise during the 8-week bed rest. The serum samples were obtained 2 days before the study (baseline), in the bed rest period (BR) and in the following recovery period (R). The serum samples were stored at -80°C until further measurement. The muscle mass of both groups were assessed by MRI and DXA during the three periods. The study was approved by the ethics committee of the Charité Universitätsmedizin Berlin. Subjects gave their written informed consent.

### Antibody development for Pro-C6

We used the last 10 amino acids of the collagen type VI α3 chain (^3168’^KPGVISVMGT^’3177^, Chinese Peptide Company, China) as the immunogenic peptide to generate specific monoclonal antibodies. All the work on mice was approved by Beijing laboratory animal administration office and animal ethics committee of Nordic Bioscience (Beijing). The methods used for monoclonal antibody development were as previously described [28]. Briefly, 4-6-week-old Balb/C mice were immunized subcutaneously with 200μl emulsified antigen with 60μg of the immunogenic peptide. Consecutive immunizations were performed at 2-week intervals in Freund's incomplete adjuvant, until stable sera titer levels were reached, and the mice were bled from the 2nd immunization on. At each bleeding, the serum titer was detected and the mouse with highest antiserum titer and the best native reactivity was selected for fusion. The selected mouse was rested for 1 month followed by intraperitoneal boosting with 50μg of immunogenic peptide in 100μl 0.9% sodium chloride solution 3 days before isolation of the spleen for cell fusion.

The fusion procedure has been described elsewhere [29]. Briefly, mouse spleen cells were fused with SP2/0 myeloma fusion partner cells. The fusion cells were raised in 96-well plates and incubated in the CO2-incubator. Here standard limited dilution was used to promote monoclonal growth. Cell lines specific to the selection peptide and without cross-reactivity to neither elongated peptide (KPGVISVMGTA, Chinese Peptide Company, China) nor truncated peptide (KPGVISVMG, American Peptide Company, USA) were selected and sub-cloned. At last the antibodies were purified using an IgG column.

### Pro-C6 assay protocol

ELISA-plates used for the assay development were Streptavidin-coated from Roche (cat.: 11940279). All ELISA plates were analyzed with the ELISA reader from Molecular Devices, SpectraMax M, (CA, USA). We labeled the selected monoclonal antibody with horseradish peroxidase (HRP) using the Lightning link HRP labeling kit according to the instructions of the manufacturer (Innovabioscience, Babraham, Cambridge, UK). A 96-well streptavidin plate was coated with biotinylated synthetic peptide biotin-KPGVISVMGT (Chinese Peptide Company, China) dissolved in coating buffer (40 mM Na_2_HPO_4_, 7 mM KH_2_PO_4_, 137 mM NaCl, 2.7 mM KCl, 0.1% Tween 20, 1% BSA, pH 7.4) and incubated 30 minutes at 20°C. 20 μL of standard peptide or samples diluted in incubation buffer (40 mM Na_2_HPO_4_, 7 mM KH_2_PO_4_, 137 mM NaCl, 2.7 mM KCl, 0.1% Tween 20, 1% BSA, 5% Liquid II, pH 7.4) were added to appropriate wells, followed by 100 μL of HRP conjugated monoclonal antibody 10A3, and incubated 21 hour at 4°C. Finally, 100 μL tetramethylbenzinidine (TMB) (Kem-En-Tec cat.438OH) was added and the plate was incubated 15 minutes at 20°C in the dark. All the above incubation steps included shaking at 300 rpm. After each incubation step the plate was washed five times in washing buffer (20 mM Tris, 50 mM NaCl). The TMB reaction was stopped by adding 100 μL of stopping solution (1% H_2_SO_4_) and measured at 450 nm with 650 nm as the reference.

### Pro-C6 technical evaluation

The lower limit of detection (LLOD) was determined from 21 zero samples (i.e. buffer) and calculated as the mean + 3x standard deviation. Upper limit of detection (ULOD) was determined as the mean– 3xSD of 10 measurements of Standard A. The intra-assay and inter-assay variation was the mean variations of 8 QC samples run 12 independent times in duplicate. Dilution recovery was determined in 4 serum samples and 4 heparin plasma samples and was calculated as a percentage of recovery of diluted samples from the 100% sample. Correlation between serum and plasma was determined in serum and the matched heparin plasma, citrate plasma, EDTA plasma from 16 individuals (Innovative Research).

### Measurement of Pro-C3, C6M assays in Berlin bed rest study

The protocols of Pro-C3, C6M assays have been described elsewhere [14, 23]. Briefly, in Pro-C3 assay, a 96-well streptavidin plate was coated with biotinylated synthetic peptide and incubated 30 minutes at 20°C. 20 μL of standard peptide or 1:2 diluted serum samples were added to appropriate wells, followed by 100 μL of HRP conjugated monoclonal antibody NB61N-62, and incubated 20 hour at 4°C. Finally, 100 μL TMB was added and the plate was incubated 15 minutes at 20°C in the dark. The TMB reaction was stopped by adding 100 μL of stopping solution (1% H_2_SO_4_) and measured at 450 nm with 650 nm as the reference. In C6M assay, biotinylated synthetic peptide is coated to a 96-well streptavidin plate. 20 μL of standard peptide or 1:2 diluted serum samples are added, followed by 100 μL of HRP conjugated monoclonal antibody, and incubated 1 hour at 20°C. The plate was read after the development by TMB.

### Statistics

The biomarker data measured in the Berlin Bed Rest clinical study were initially subjected to a distribution analysis in order to determine of transformation was necessary to obtain prerequisite normality. Then the data were subjected to mixed models repeated measures ANOVA analysis, testing for Time and Treatment (CTRL vs. RVE) effects as well as Time*Treatment interactions. This was done in the first instance for the entire Time Course, and then for the time points covering the remobilization only (starting with the last immobilization time point). If main effects were present, appropriate post hoc tests were performed, subjected to Bonferroni correction for multiple testing. The correlation analyses between biomarkers and LBM were performed with Graphpad Prism as a linear regression, yielding (parametric) Pearson correlation coefficients. Significance threshold is determined to be p<0.05 and data are presented as means ± SEMs where appropriate.

## Results

### Characterization of Pro-C6 assay

**Fig 1:**
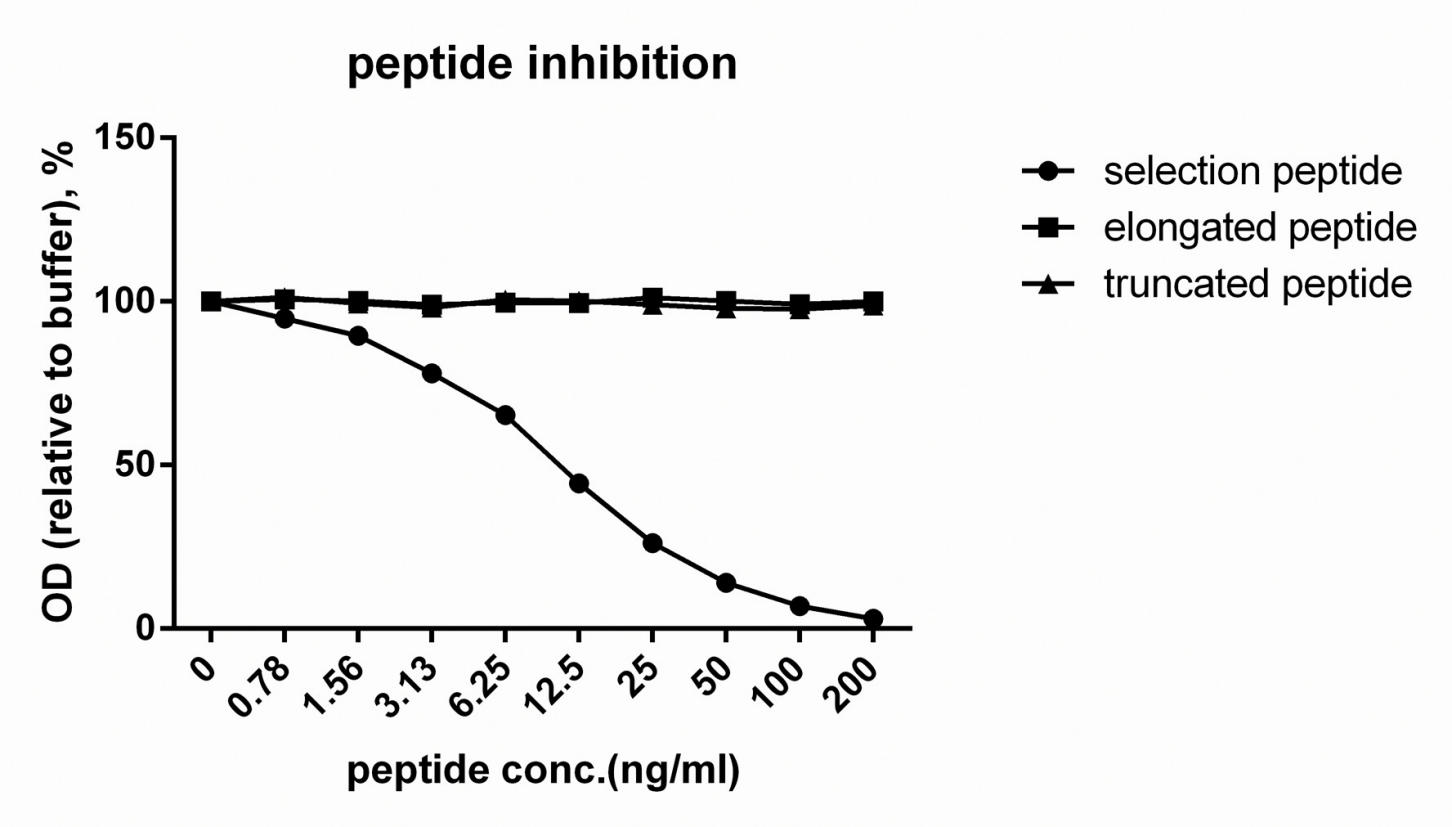
Peptide specificity test of monoclonal antibody 10A3. The antibody only recognized the selection peptide, and has no cross reaction to neither elongated peptide nor truncated peptide.

**Fig 2:**
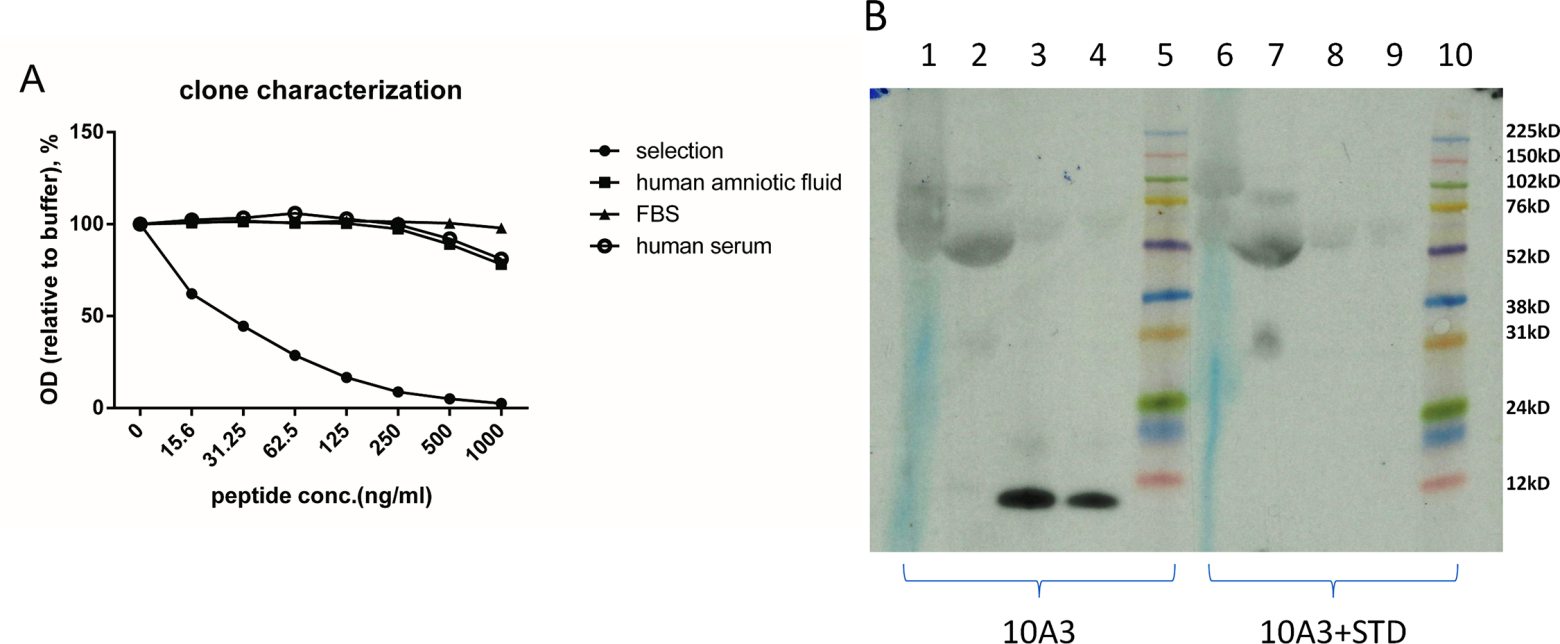
Pro-C6 reacted with human serum and amniotic fluid. (A) The signals in competitive ELISA were partly inhibited by human serum and human amniotic fluid. (B) Western blot showed the specific bands in human serum (lane 1, 2) and amniotic fluid (lane 3, 4). The bands can be blocked in the presence of selection peptide (lane 6–9).

The chosen antibody 10A3 specifically recognized the last 10 amino acids of C-terminus COL6A3 ^3168^’KPGVISVMGT’^3177^, but did not recognize elongated peptide KPGVISVMGTA or truncated peptide KPGVISVMG (Fig 1). Native reactivity of the chosen antibody was assessed using human serum pool and human amniotic fluid pool. In the competitive ELISA, the signals were partly inhibited by both serum and amniotic fluid (Fig 2A). Western blot showed that the antibody recognized a band around 10kD, a signal which was completely blocked in the presence of the standard peptide (Fig 2B).

**Fig 3:**
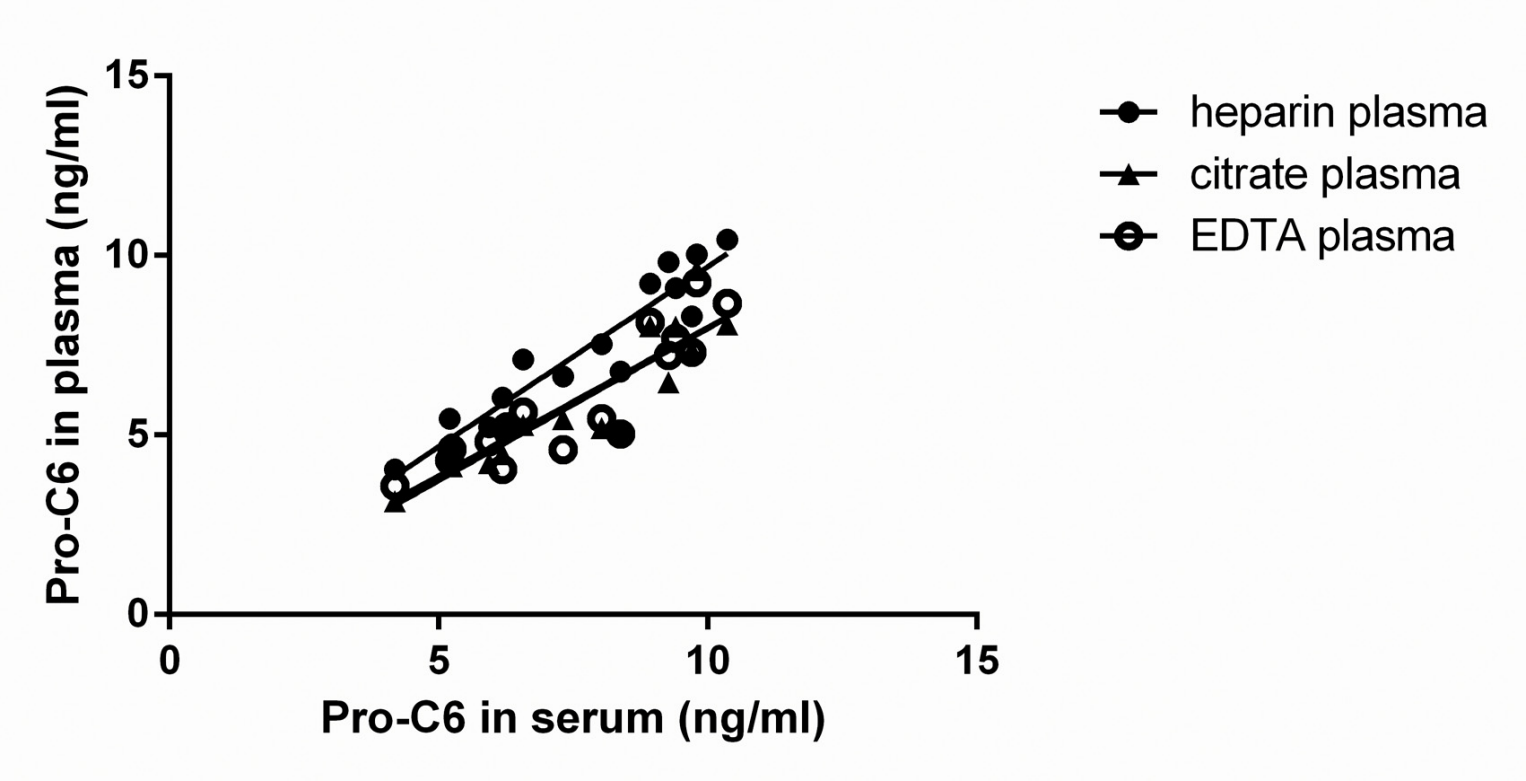
Correlation of Pro-C6 levels in three kinds of plasma and serum (matched samples, from 16 individuals). Linear regression analysis was performed, plasma vs serum. The figure shows the strong correlations of Pro-C6 levels in serum and the three kinds of plasma (P<0.0001).

The measurement range of the Pro-C6 competitive ELISA was determined providing a range from 0.15ng/ml (LLOD) to 58.39ng/ml (ULOD). The inter- and intra- assay variability are 15.2% and 4.8%, respectively. The dilution recovery in human serum and heparin plasma were both within 100±20% (Table 1). The correlations between values in human serum and three kinds of plasmas were relatively high (Fig 3, P<0.0001), showing that Pro-C6 levels are independent of blood preparation method.

**Table 1:** Dilution recovery of Pro-C6 assay.

Serum samples	Dilution recovery	Heparin plasma samples	Dilution recovery
**undiluted**	100	**undiluted**	100
**dilution 1:2**	91	**dilution 1:2**	105
**dilution 1:4**	91	**dilution 1:4**	100
**dilution 1:8**	80	**dilution 1:8**	109

Notes: Samples were diluted in serial 2-fold dilution steps concentration was measured in these serial dilutions. Dilution recovery was obtained by multiplying measured concentrations with the dilution factor and expressed as percent of the concentration of the undiluted (starting) sample. The table shows that the signal dilutes linearly and stays within +/- 20% within and 8-fold dilution range.

### Pro-C6 biomarker profile in Berlin Bed Rest study

**Fig 4:**
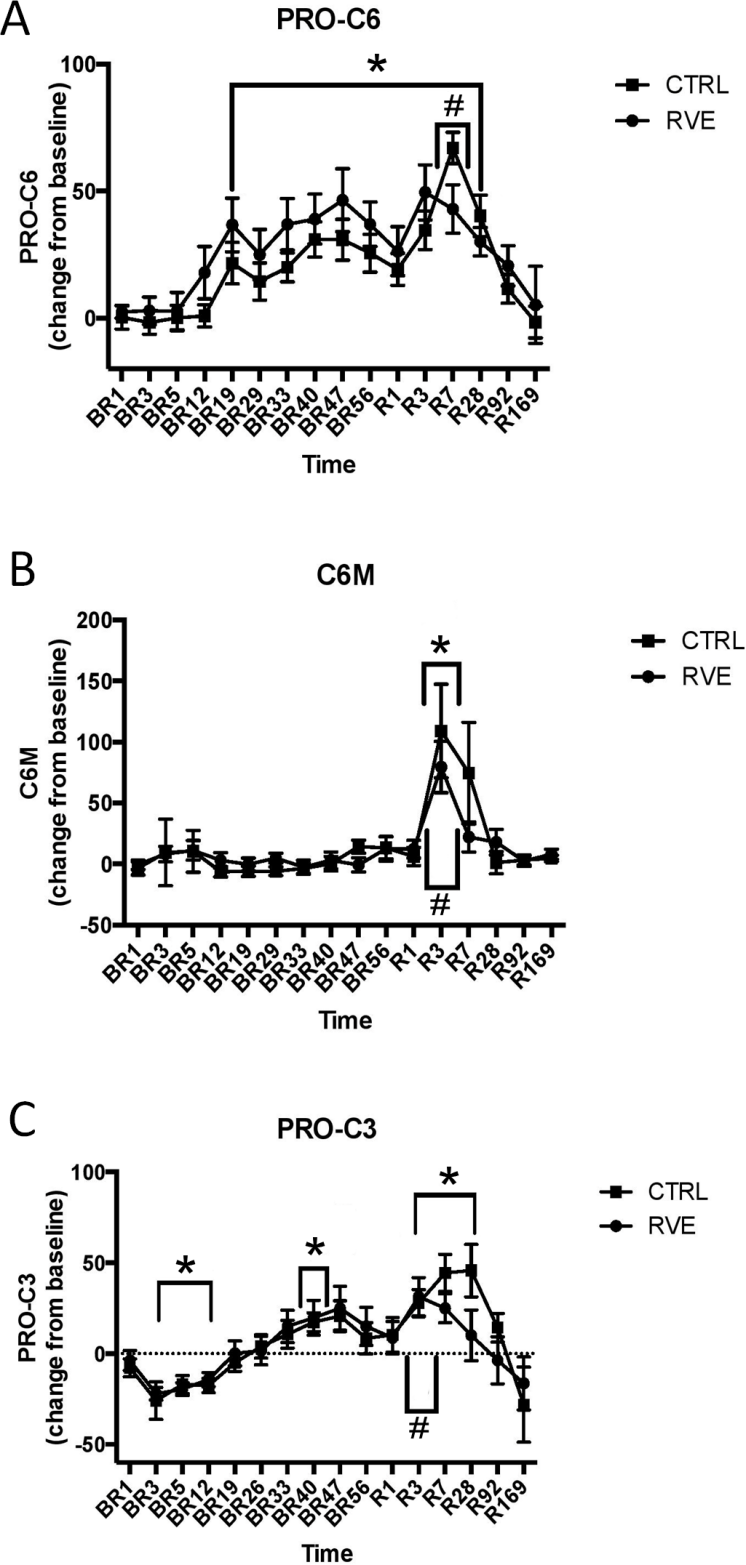
Biomarker changes relative to baseline (A, B, C) in the BBR1 study. (A) Pro-C6 changes relative to baseline (%). (B) C6M changes relative to baseline (%). (C) Pro-C3 changes relative to baseline (%). “BR” time points denote bed rest immobilization time points and “R” time points denote remobilization time points. The number suffix denotes the number of days into the bed rest or remobilization period. “*” denotes the days that have significantly different levels when compared to baseline, p<0.05. “#” denotes significant difference in the level when compared to the last time point of the immobilization period (BR56), p<0.05. Data are expressed as means ±SEMs.

The Pro-C6 biomarker (Fig 4A) changed over time during the course of immobilization (significant time effect, p<0.0001) in the form of an increase after approximately one week of immobilization, reaching a peak level approximately 40% higher than baseline during the last couple of weeks of immobilization (being significantly higher than baseline from BR19 to R28, peaking at BR47, p = 0.0002). There were no differences between the RVE and CTRL group during the immobilization period (no significant treatment effects or treatment*time interactions).

During re-mobilization, both time and time*treatment interaction effects manifested themselves. This was in the form of an increase that peaked one week into remobilization (a 20% increase relative to the last day of immobilization, BR56, p = 0.011), followed by a gradual return to baseline values. The interaction effect was not manifested in any post hoc tests, owing to high variation at the R7 time point.

When we compared individual biomarker levels of Pro-C6 with LBM and changes therein (Table 2), we found that the level of Pro-C6 at baseline was not related to LBM at all, but the level at BR47 was positively related to change in LBM during immobilization (R^2^ = 0.2794, R = 0.529, p = 0.0166) meaning that higher levels of Pro-C6 were associated with lower loss of LBM. We also found that Pro-C6 at R3 was negatively related to the amount of LBM (re)gained during remobilization (R^2^ = 0.3365, R = 0.580, p = 0.0073), meaning that higher levels were associated with less (re)gain of LBM during remobilization.

**Table 2:** Correlation matrix for biomarker vs. anthropometric variables.

	Pro-C6		C6M		Pro-C3	
	R	p	R	p	R	p
**BioM**_**Baseline**_ **vs. LBM**_**Baseline**_ **	0.022	0.9270	0.595	0.0057*	0.536	0.0149*
**BioM**_**BR47**_ **vs. leg LBM**_**Loss**_	0.529	0.0166*	0.102	0.6684	0.453	0.0447*
**BioM**_**R3**_ **vs. leg LBM**_**Gain**_	-0.580	0.0073*	-0.269	0.2509	-0.171	0.4705

Notes

*) represent significant difference

**) BioM (Biomarker), Lean Body mass (LBM). Loss is the absolute LBM change during immobilization, i.e. higher negative equals bigger loss; Gain is total LBM regain during remobilization.

### C6M biomarker profile in Berlin Bed Rest study

The C6M biomarker (Fig 4B) was essentially unchanged during the immobilization phase (no time effect in the immobilization time period), but increased briefly 30–40% at the beginning of remobilization (a significant time effect at p<0.0001 during the immobilization period). There were no effect of RVE treatment during immobilization and although it may appear as if the increase in the C6M signal is bigger in the CTRL group than in the RVE group, this did not reach significance (the time*treatment interaction did not reach significance and thus no post hoc test was made).

When we compared individual biomarker levels of C6M with LBM and changes therein (Table 2), we found that the level of C6M at baseline correlated with the baseline LBM (R^2^ = 0.3540, R = 0.595, p = 0.0057), but not related to muscle loss during immobilization at BR47 nor to the amount of muscle (re)gained during remobilization at R3.

### Pro-C3 biomarker profile in Berlin Bed Rest study

Pro-C3 (Fig 4C) displayed a significant time effect in the form of an initial decrease of approximately 20% upon immobilization (being significantly different from baseline from BR3 through BR12, p<0.004 for all time points) followed by an increase at the end of the immobilization (BR40 being significantly different from baseline, p = 0.05). Interestingly, at the onset of remobilization, a similar pattern could be observed with an initial decrease followed by an increase (with time points R3 through R28 being significantly higher than baseline, p<0.03 for all time points, and R3 being significantly higher than the last time point of immobilization, BR56, p = 0.02). At the last two time points, 13 weeks following the onset of immobilization, the biomarker levels were back to baseline. There were no significant between-group differences, nor a significant time*treatment interaction effect.

When we compared individual biomarker levels of Pro-C3 with LBM and changes therein (Table 2), we found that individual levels of Pro-C3 correlated significantly with LBM at baseline (R^2^ = 0.2869, R = 0.536, p = 0.0149). Furthermore, we found that the level of biomarker at its peak at BR47, correlated significantly with the amount of LBM lost during immobilization (R^2^ = 0.2056, R = 0.453, p = 0.0447).

## Discussion

Immobilization leads to loss of the lean body mass, predominantly in the form of skeletal muscle, as was also the case for the present study (study previously described in detail, including detailed changes in body composition) [27, 30]. Stable isotope studies have shown that atrophy in most tissues and muscle in particular is driven by global decreases of synthesis to a larger extent than increases in degradation [31, 32].

There is little research on the effect of immobilization on muscle ECM turnover on proteome scale as well as for individual proteins. Naturally, turnover of individual proteins can be regulated in a different manner. Looking at rodent studies, the total muscular collagen was increased during immobilization [33]. Of particular interest, in the Miller study, immobilization resulted in increases in collagen III protein [33], which occurs in despite of down-regulated collagen III mRNA reported elsewhere in rat muscle unloading in a similar model [34].

In this paper, we report the modulation of serological collagen peptide biomarkers in response to long-term unloading in the form of bed rest and subsequent reloading in the Berlin Bed Rest study. In this study, the subjects were immobilized through bed rest with or without a vibration device countermeasure for 8 weeks followed by remobilization through habitual physical activity. Both groups lost muscle mass and strength during the immobilization, with slightly more lost in the CTRL group than in the RVE group. Both group regained muscle mass and strength during remobilization.

During immobilization, the biomarkers Pro-C3 and Pro-C6 display somewhat similar temporal patterns. While Pro-C3 initially drops slightly following the onset of immobilization, both Pro-C3 and Pro-C6 eventually increase with immobilization over time, 20% and 40% respectively. At the onset of remobilization, a slight initial drop can again be observed followed by an increase that on the part of Pro-C6 was bigger in the CTRL than in the RVE group, followed by a return to baseline in both biomarkers. The C6M biomarker is essentially unresponsive to bed rest unloading, but spikes briefly in response to reloading, with no significant difference between groups.

Collagen type III is expressed ubiquitously in tensile stress bearing tissues, including, but not restricted to, tendon, muscle, vasculature and skin. This essentially restricts expression to Lean Body Mass. We have in two previous studies shown that baseline levels of the Pro-C3 biomarker correlate with LBM in healthy adults [14, 35]. We believe that the measured levels of the biomarker reflect the total level of collagen III steady-state synthesis and thus, by extension, the amount of LBM.

The finding from the Miller study fits well with the temporal Pro-C3 pattern we observed, as the Pro-C3 is a biomarker of collagen III synthesis [36]. The further increase in Pro-C3 we observed during remobilization, also matches well with findings from both animal and human studies. These have shown increases in collagen protein synthesis and content [36, 37].

While it can appear counterintuitive that counter-directional stimuli produce the same biomarker response, we can only speculate that both the detrained and more trained phenotypes are associated with specific ECM mechanical and compositional profiles that each requires specific remodeling, thus explaining the changes observed.

Collagen VI is ubiquitously expressed basement membrane protein expressed as heterotrimers of alpha1, alpha2 and either alpha3, alpha5 or alpha6 chains (the alpha4 is absent in humans). However, it appears to be particularly important or at least partially indispensable in skeletal muscle as genetic defects in α1(VI), α2(VI) or α3(VI) chains result in dystrophic phenotypes. In these dystrophic phenotypes creatine kinase is chronically elevated, indicating loss of sarcolemma integrity [20]. This matches well with the supposed function in which Collagen VI surrounding cells, help anchor them into the surrounding extra-cellular matrix.

When it comes to discussing the changes in Collagen type VI biomarkers it becomes more difficult as these are very poorly described in relation to changes in physical activity. There is essentially no information about collagen type VI expression at mRNA or protein levels in homogenates or histochemistry in healthy human muscle. Our interest in peptides derived from Collagen type VI were derived from previous studies, where we found that a biomarker derived from the α1(VI) chain correlated well with LBM in healthy adults [38] and the fact that Collagen type VI appears indispensable in skeletal muscle, as mutations in these chains cause Ullrich and Bethlem Myopathies. The temporal pattern of Pro-C6 during the course of immobilization and subsequent remobilization is similar to Pro-C3.

In muscle it has been shown that the abundance of Collagen type III relative to total collagen is higher in endomysium, the connective tissue sheath surrounding individual muscle fibers. Considering that Collagen type VI is a basement membrane protein, their shared pattern could indicate that protein turnover of the proteins making up the muscle fiber niche are regulated in a parallel manner.

The correlation analysis revealed that higher levels of Pro-C6 were associated with smaller loss of muscle during immobilization and higher levels were also associated with a smaller degree of muscle (re)gain during remobilization. While it may appear counterintuitive, this may be confounded by the ones that lost the most LBM also regained the most. Collagen type VI is involved in proper satellite cell function [39] and autophagic function [40], so while there is plenty of possibly causal and/or mechanistic relationships explaining the temporal pattern we see, at the present there is inadequate knowledge available for us to suggest which ones might be at play.

Collagen type III and type VI have different structure and functions in muscle. Collagen type III is a fibrillar collagen, usually co-distributes with collagen type I. While Collagen type VI forms a unique microfibrillar network and acts as a linker between the cell skeleton and ECM. Therefore, their remodeling rate in immobilization and remobilization could be different, which could be observed in the specific biomarker responses. C6M is unchanged during muscle atrophy, and only spikes in the early phase of remobilization, shows some similarity of creatine kinase [41]. While Pro-C3 shows an initial drop in the early phase of immobilization, then Pro-C3 and Pro-C6 increase during the atrophy and spike in remobilization.

It is worth noting that none of the biomarkers display significantly different patterns between the two treatments in the study. This can most likely be attributed to the fact that the changes in lean mass with the study were not as large as those reported for total immobilization, e.g. from casting. There are significant differences of muscle volume change in some lower limb (from MRI) between two groups [27]. We can also observe a loss of 8% LBM (from DXA) in the lower body in the CTRL group and 4% in the RVE group, however, there is no change or even a small increase in upper body LBM [30]. As these biomarkers are presumably produced during connective tissue turnover and released to the circulation, this differential muscle response translates into a mixed signal in serum, thereby explaining a limited sensitivity to the different interventions.

Although there is no significant difference between two treatments, Pro-C3, Pro-C6 and C6M appear to have greater increase in control group, and Pro-C3 and C6M last longer to return to baseline. This finding might reflect greater muscle loss in control group compared with RVE group in some of the major postural muscles [27] and potentially more muscle damage [41], which leads to the needs for more muscle re-building.

With increased age or systemic pathologies, fibrogenicity in all tissues increases and eventually some degree of fibrosis also manifests in muscle. The increase in propeptide biomarkers observed here, could be understood in that context, i.e. that muscle fibrosis in the elderly or the seriously ill, is not driven by age or pathology *per se*, but by repeated cycles of immobilization. This would parallel the proposed mechanism for age-related muscle loss, which has also been suggested to be driven by discrete catabolic events, rather than a sustained catabolic or anti-anabolic drive [42].

While this is obviously hypothetical, immobilization in animal models have also been shown to lead to intramuscular fibrosis [43, 44].

As these peptide biomarkers are released to the circulation and expressed in many different tissues it is difficult to prove the exact origin of them. In this paper we are in part just reporting the temporal patterns and in part comparing them with changes in LBM, in the context of a bed rest immobilization and remobilization study.

We cannot exclude the possibility that the biomarkers correlations we report are driven by other effects, e.g. changes in hepatic circulating peptide clearance. Also, we must appreciate the fact that several processes can contribute to production of biomarker peptides. For example, even though we report here (and have done so before) that in healthy adults, Pro-C3 correlates with LBM [14, 38], it has also been shown that other processes increases the amount of collagen III propeptide in the circulation, like liver fibrosis or anabolic drug administration [12, 14, 45]. Therefore the inferences made about the biomarkers should only be generalized to other populations with consideration of this.

There is very little scientific literature about the changes in collagen expression and metabolism with unloading and reloading and most of the literature that does exist focuses on collagens I and III using histochemistry, mRNA expression or analysis of hydroxyproline content. While these findings may help interpreting our collagen III results, there is practically no scientific literature about collagen type VI and collagen type VI peptides, making contextualizing our findings there very limited.

## Conclusion

Immobilization is associated with pronounced modulation of Pro-C3 and Pro-C6, but not C6M. The upregulation of both Pro-C3 and–C6 likely reflects remodeling of the extracellular matrix in muscle in response to unloading and reloading. Pro-C6 was related to the anabolic and catabolic responses to unloading and reloading. Furthermore, we can report that Pro-C3 is correlated with LBM in young healthy adults, extending previous findings from our group.
